# Development of a framework and research impact capture tool for nursing, midwifery, allied health professions, healthcare science, pharmacy and psychology (NMAHPPs)

**DOI:** 10.1186/s12913-023-09451-2

**Published:** 2023-05-03

**Authors:** Lisa Newington, Mary Wells, Samina Begum, Andy J. Lavender, Sarah Markham, Oliver Tracy, Caroline M. Alexander

**Affiliations:** 1 Department of Surgery and Cancer, Faculty of Medicine, Imperial College London, London, UK; 2 Imperial College Healthcare NHS Trust, London, UK; 3 Patient and Public Advisory Group Member, London, UK

**Keywords:** Nursing, Midwifery, Allied health professions, Healthcare science, Pharmacy, Psychology, NMAHPP, Research impact, Clinical academic, Patient and public involvement

## Abstract

**Background:**

There is an ambitious target to create a UK clinical academic workforce representing 1% of clinicians from nursing, midwifery, the allied health professions, healthcare science, pharmacy and psychology (NMAHPPs). Understanding and recording the impact that clinical academics make across healthcare services is crucial if we are to grow, value and support this highly skilled workforce group. However, it is currently difficult to systematically record, collate and report the impacts associated with NMAHPP research activity. The aims of this project were to i) develop a framework outlining the impacts that were important for key stakeholder groups, and ii) create and pilot a research impact capture tool to record these impacts.

**Methods:**

The framework was developed from the existing literature. It was refined, remodelled and approved by multidisciplinary stakeholder involvement, including patient and public representatives, healthcare managers and research-active clinicians. The framework was converted into a series of questions to create an electronic research impact capture tool, which was also refined through feedback from these stakeholder groups. The impact capture tool was piloted with research-active clinicians across a large NHS Trust and its associated organisations.

**Results:**

The impact framework contained eight elements: clinical background, research and service improvement activities, research capacity building, research into practice, patients and service users, research dissemination, economics and research funding, and collaborations. Thirty individuals provided data for the research impact capture tool pilot (55% response rate). Respondents reported a range of positive impacts representing all elements of the framework. Importantly, research-activity appeared to be a key driver for recruitment and retention in the sample population.

**Conclusions:**

The impact capture tool is a feasible method of recording the breadth of impacts associated with NMAHPP research activity. We encourage other organisations to collaboratively use and refine our impact capture tool, with the aim of standardising reporting, and facilitating discussions about research activity within clinical appraisal. Pooling and comparing data will also allow comparison between organisations, and assessment of change over time or after implementation of interventions aimed at supporting and increasing research activity.

**Supplementary Information:**

The online version contains supplementary material available at 10.1186/s12913-023-09451-2.

## Background

Increasing research activity by clinicians from nursing, midwifery, the allied health professions (Supplementary file 1), healthcare science, pharmacy and psychology (NMAHPPs) is a key objective for the UK health service [1–5]. At the level of the healthcare organisation, research activity has been linked with better patient outcomes, including increased satisfaction [6], decreased mortality [7] and improved healthcare performance [7, 8]. However, there is currently a lack of parity between research roles for clinical doctors and those for NMAHPP clinicians, despite the latter forming the large majority of the healthcare workforce [9]. Approximately 5% of UK medical consultants are employed in clinical academic roles, i.e. roles that combine clinical practice with research activity and research leadership [10, 11]. There are also several clinical academic opportunities for junior doctors [11]. For NMAHPPs, the target is to achieve a clinical academic workforce of 1% by 2030 from a starting point of 0.1% in 2010 [12].

At the individual level, the scarcity of clinical academic roles, a perceived lack of value of academically trained NMAHPPs, and the absence of clear clinical academic leadership were all reported as key barriers for those wishing to pursue a clinical academic career [13–17]. Key enablers included access to support and guidance, and securing external funding [13–15, 17].

There is widespread recognition that NMAHPP research leadership is essential to support the establishment of relevant research, research capacity building, and to support the implementation of research findings into practice [12, 18–21]. As NMAHPP research activity increases, there is also a need to record and evaluate the spectrum of impacts that are realised through this research activity. Large research funding bodies frequently use self-reported impact capture methods, such as ResearchFish® to collect impact data [22–24], however this information is not accessible to be pooled across different awardees on unlinked projects working within the same healthcare organisation. Furthermore, not all research activity is funded by these bodies or reported via this system.

For organisations striving to increase and support NMAHPP research activity, demonstrating the value of this activity is crucial. However, it is difficult to systematically record and report the variety of impacts associated with this investment. A recent UK annual survey conducted by the Clinical Academic Research Careers Implementation Network (CARIN) found that only two of the 37 responding NHS organisations collected any data on research impact (Mary Wells, personal communication 12/12/2022). In addition, different stakeholder groups may have different views on the value of individual measures of impact [25]. Our previous work documented the types of impact that were associated with NMAHPP-led healthcare research as reported in the literature [26]. We also explored the views of healthcare managers, research-active clinicians [27] and people who had been participants or advisors for NMAHPP-led health research [28]. The current work took place in the context of a large NHS Trust that had a pre-existing research strategy for NMAHPP professions [29] and offers locally funded competitive research fellowships [30], in addition to supporting NMAHPP applications for national research funding schemes.

The aims of the current project were (i) to develop a framework that integrates the impacts of NMAHPP research activity as identified by different stakeholder groups, and (ii) to create and pilot a research impact capture tool that could be used to document these impacts.

## Methods

### Development of the framework

Framework development followed the three stages outlined by McMeekin et al.: i) identifying relevant data, ii) developing the framework, iii) validating, testing and refining the framework [31].

Relevant data related to the reported impacts of research activity by NMAHPPs. This included our previous systematic review [26], qualitative interviews with healthcare managers and research-active clinicians [27] and qualitative interviews with research participants and patient advisors [28]. Additional evidence was identified using a targeted literature search limited to the period after the previous systematic review (January 2020 to October 2022). The newly identified literature primarily focused on clinical academic careers and developing research capability and capacity [13, 17, 19, 20, 32–38].

To develop the framework, the findings from our existing research were pooled and organised by the authors to create key impact themes. These were cross referenced with data identified in the additional literature. The preliminary framework was refined, validated and tested by the patient and public advisory group (PAG), the NHS Trust Clinical Academic Research Committee (CARC) and the NHS Trust Postgraduate Research Forum (PGRF). Four PAG members were recruited nationally, and all had experience as NMAHPP research participants and/or research advisors. These individuals were reimbursed for their time. CARC consists of research and clinical leads covering pharmacy, psychology, healthcare science and allied health professions within the Trust and linked organisations. The PGRF is a group of research-active NMAHPP clinicians who are affiliated with or working at the Trust and involved in research that is supported by various funding sources.

Table 1 shows the basic framework that was created through discussion, refinement and review. The reasoning behind the inclusion of each element is presented, along with the evidence used to inform and support inclusion.

**Table 1:** NMAHPP Research impact capture framework

Framework element		Reason for inclusion	Link with existing research
	**Clinical background**	Monitor research engagement and access to research opportunities for all NMAHPP disciplines and settings, and across clinical grades Record career aspirations Enable comparison of all impacts across disciplines or settings, where relevant	Visibility and accessibility of clinical academic opportunities and career pathways [13, 17, 19, 20, 26, 27, 32, 34, 36] Healthcare staff recruitment and retention [13, 26, 37]
	**Research and service improvement activities**	Identify the breadth of research and other service improvement activities, and how research-active NMAHPPs guide service improvement Record how these activities were combined with clinical roles	Balancing clinical and academic roles [13, 19, 20, 26, 32, 36]
	**Research capacity building**	Identify activities that support others to engage with research, and the outputs of this research engagement	Improvements in approach to patient care [27] Development of research culture and capabilities [19, 27, 32–35]
	**Research into practice**	Identify translation of individual research findings into practice, including the geographical reach of changes to practice	Creation and implementation of new evidence [26] Connecting health research with healthcare [28]
	**Patients and service users**	Record the nature and scope of patient and service user involvement and the influence of this on the research processes and outcomes Record how study findings are communicated with participants and other contributors	Optimising research experiences for participants [28] Improving patient care [38]
	**Research dissemination**	Record research dissemination methods, including non-traditional formats Identify the potential reach of these dissemination activities	Knowledge exchange [17, 26]
	**Economics and research funding**	Record new research funding applications and successes Identify the financial and reputational value	Financial impacts [13, 26, 28]
	**Collaborations**	Record new research collaborations and other networks, including the wider clinical and research teams involved	Development of collaborations and networks [19, 26, 32]

*NMAHPP* nursing, midwifery, allied health professionals, healthcare science, pharmacy and psychology

### Development of the impact capture tool

This framework was translated into a series of questions to capture each element. Questions and response options were reviewed by the three stakeholder groups discussed above (PAG, CARC and PGRF) and refined based on this feedback. The questions were incorporated into an online research impact capture tool (Qualtrics XM) and pilot tested within the NHS Trust with healthcare managers from the NMAHPP professions, research-active clinicians at pre-doctoral, doctoral and post-doctoral levels and PAG members. Further refinements were made at this stage and the questions were linked back to the framework elements to ensure that all aspects were included.

The pilot research impact capture tool is available via the Open Science Framework [39]. All questions were framed in relation to research activity and impact within the previous 12 months. This period was chosen to facilitate accurate recall and to enable the questions to be repeated annually to monitor change over time.

### Data collection

Approximately 55 research-active clinicians within or affiliated with the Trust were invited to complete the questions by email. Individuals known to be involved in research were contacted using existing databases and the leads for each of the NMAHPP professions were also asked to disseminate the invitation using their own records of research-active individuals. Up to two reminder emails were sent.

### Data analysis

Responses were analysed using Stata (version 15.1, StataCorp LLC). Additional comments were reviewed by the research team and grouped into similar themes.

## Results

### Respondent clinical background and demographics

The call for responses was open during November 2022. Thirty individuals responded from the approximate total of 55, yielding an estimated maximum response rate of 54.5%. Respondent demographics are provided in Table 2. All respondents were registered healthcare professionals. Respondents were asked about their academic qualifications and were categorised as ‘pre-doctoral’ if their highest qualification was at bachelor’s or master’s level, ‘doctoral’ if they were currently working towards a PhD or professional doctorate, and ‘post-doctoral’ if they had completed a PhD or professional doctorate. The majority of those at NHS band 5–6 (entry grade and first senior level) were pre-doctoral (63%), and the majority of those at band 7-8a (clinical specialist or local team lead) were currently undertaking a PhD or clinical doctorate (50%). All individuals working at NHS band 8b and above (department lead or consultant clinician) were at a post-doctoral level, however there were individuals who had completed a PhD or professional doctorate across all clinical grades.

**Table 2:** Respondent demographics

	**Respondents** *n* = 30 (%)
**Age (years)**	
18–24	1 (3)
25–34	5 (17)
35–44	10 (33)
45–54	7 (23)
55–64	1 (3)
Not reported	6 (20)
**Gender**	
Male	1 (3)
Female	23 (77)
Not reported	6 (20)
**Ethnicity**	
Asian / Asian British	2 (7)
Black / Black British	1 (3)
White / White British	17 (57)
Mixed	3 (10)
Other	1 (3)
Not reported	6 (20)
**Clinical background**	
Nurse / midwife	12 (40)
Allied health professional	15 (50)
Healthcare scientist	0
Pharmacy staff	3 (10)
Psychologist	0
**Median years post clinical qualification [range]**	
Nurse / midwife	10 [3-27]
Allied health professional	16 [2-38]
Pharmacy staff	12 [5-31]
**Academic level**	
Pre-doctoral (any stage before PhD)	9 (30)
Doctoral (undertaking a PhD or professional doctorate)	10 (33)
Post-doctoral (completed a PhD or professional doctorate)	11 (37)
**Substantive employer**	
NHS	19 (63)
University	3 (10)
Joint NHS/University	3 (10)
Separate contracts	5 (17)
**NHS band**	
5–6 (entry grade and first senior level)	8 (27)
7-8a (clinical specialist or local team lead)	16 (53)
8b and above (departmental lead or consultant clinician)	5 (17)
Not applicable, solely based in a university	1 (3)
**Current research funding (responses not mutually exclusive)**	
Locally funded fellowship	7 (23)
Nationally funded fellowship	10 (33)
Locally funded grant	3 (10)
Nationally funded grant	6 (20)
International funding	0
Industry or commercial funding	1 (3)
Not reported	11 (37)

Half of respondents had been employed by their current organisation for more than 5 years (*n* = 15), compared with 27% (*n* = 8) for 2–5 years and 23% (*n* = 7) for less than 2 years. Duration varied according to academic level; more than 55% of doctoral and post-doctoral respondents had been at their current organisation for more than 5 years versus 33% of pre-doctoral respondents.

Those who moved to a new organisation within the last 2 years were asked to select the reasons why they chose to move. Those who had worked at the same organisation for at least 2 years were asked to select the reasons why they chose to stay. Responses are summarised in Table 3.

**Table 3:** Reported reasons for joining or remaining at current organisation

	**Reasons for joining** (< 2 years with employer) *n* = 7 (%)		**Reasons for remaining** (≥ 2 years with employer) *n* = 23 (%)	
**Opportunities for development**				
Clinical opportunities	3 (43)	^a^	10 (43)	^b^
Leadership opportunities	3 (43)		-	
Access to relevant training	4 (57)		11 (58)	
Research opportunities, including access to funding	4 (57)	^b^	20 (87)	^c^
Opportunities to be involved in teaching	-		7 (30)	
**Reputation of the individuals or organisation**				
Reputation of individual or team – clinical	2 (29)		-	
Reputation of individual or team – research	4 (57)	^a^	-	
General reputation of organisation	1 (14)	^a^	-	
**Networks**				
Links with an Academic Health Science Centre	3 (43)		11 (48)	
Access to networks and groups	0		5 (22)	
**Wellbeing**				
Work-life balance	2 (29)	^a^	6 (26)	^b^
Flexible working opportunities	2 (29)	^a^	10 (43)	^b^
Enjoy working for this organisation	-		10 (43)	
**Convenience**				
Convenient location	1 (14)		9 (39)	^a^
Didn’t know much about the organisation	0		-	
Difficult to find work elsewhere	-		5 (22)	
Haven’t considered moving elsewhere	-		5 (22)	^a^
**Support**				
Supportive team and immediate manager	-		12 (52)	^a^
Supportive organisational leadership	-		9 (39)	
Support to carryout research alongside clinical practice	-		9 (39)	
**Other**				
Ongoing research grant or fellowship	-		14 (61)	^b^
Planning on moving in next 12 months	-		2 (9)	^a^

Response options not mutually exclusive.—Question not asked

Most important reason for joining/remaining at the organisation: ^a^ selected by 1 individual, ^b^ selected by 2–5 individuals; ^c^ selected by 6–10 individuals

Respondents were also asked to identify the most important reason for joining or remaining at their organisation. For both groups, the majority selected ‘research and clinical academic opportunities, including access to research funding’ (< 2 years 29%, ≥ 2 years 40%).

Fourteen respondents (47%) reported that at least one person had applied for a role in their department within the past 12 months because of the research culture and opportunities. A further 10 (33%) were unsure.

Nearly three quarters of respondents held an honorary contract linked with their research activity (*n* = 22, 73%). The majority were NHS employees holding honorary contracts with a university (*n* = 18, 82%). The remainder were either NHS employees holding contracts with additional NHS organisations, or university employees holding an honorary contact with the NHS. Honorary titles could be classified into six groups: research fellow or clinical research fellow, research officer, research assistant or associate, clinical lecturer, consultant, and professor.

**Fig. 1:**
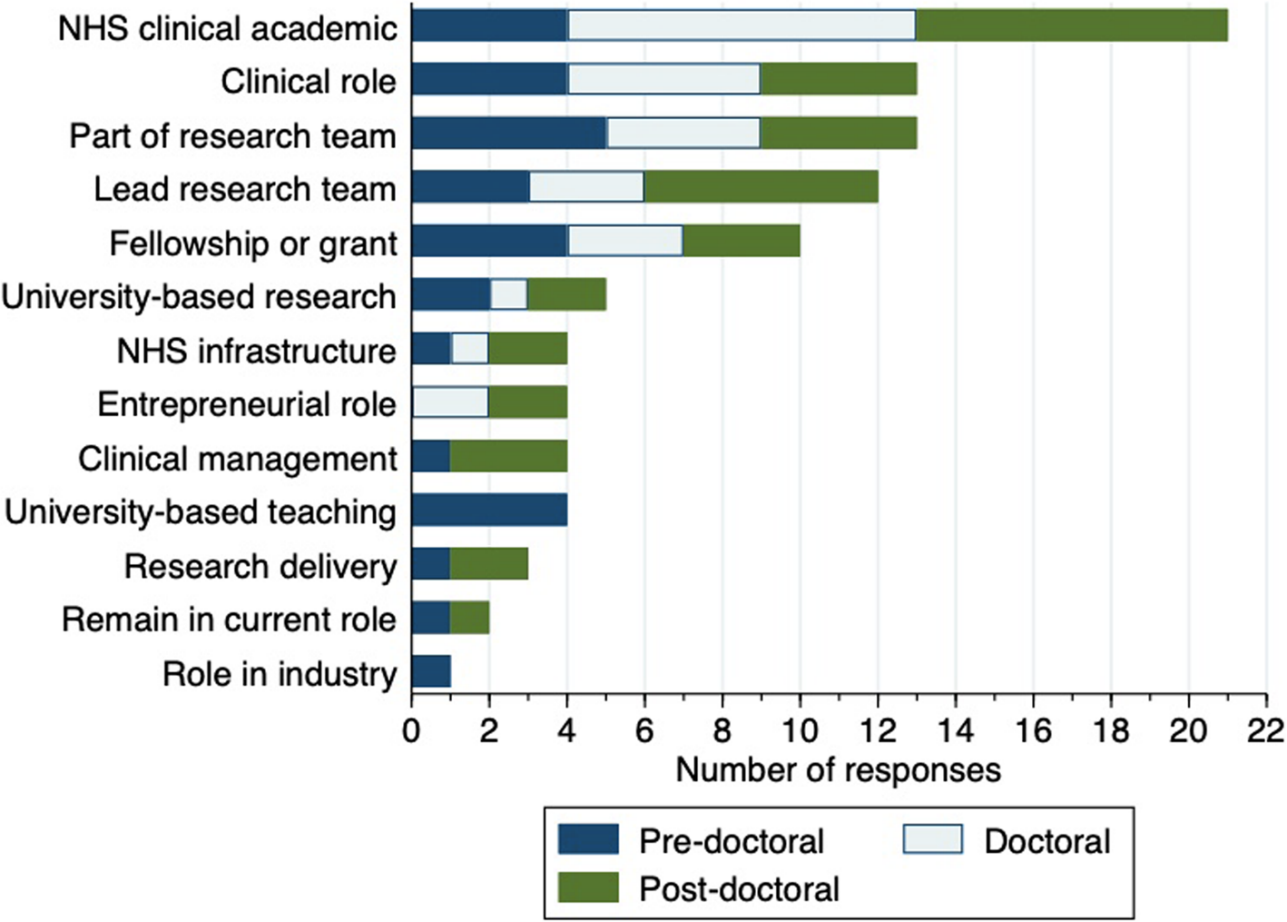
Reported ideal career step within the next 5 years

All respondents were asked about their ideal career progression within the next 5 years. The most commonly selected option was ‘clinical academic – based in the NHS’, as chosen by 21 respondents (70%; Fig. 1). Seven individuals also provided additional comments. All focused on a desire to establish combined clinical academic roles that enabled specific time for clinical activity, research capacity building, and research.

### Research and service improvement activities

Within the previous 12 months, respondents reported involvement in a combination of research, service evaluation, audit and quality improvement (QI) projects. These activities are summarised in Table 4. Compared with pre-doctoral respondents, a greater proportion of doctoral and post-doctoral respondents were involved in research leadership and related supervisory and governance roles.

**Table 4:** Reported research activities in the previous 12 months

	**Pre-doctoral** *n* = 9 (%)	**Doctoral** *n* = 10 (%)	**Post-doctoral** *n* = 11 (%)
**Research**			
Preparing an application	3 (33)	6 (60)	7 (64)
Undertaking a personal research fellowship or project	4 (44)	10 (100)	8 (73)
Collaborating on research led by NMAHPP	4 (44)	2 (20)	4 (36)
Collaborating on research led by clinical doctor or academic	3 (33)	3 (30)	2 (18)
Leading a study or programme of research	1 (11)	2 (20)	7 (64)
Formal research supervisor	1 (11)	3 (30)	6 (55)
Informal research supervisor	0	4 (40)	6 (55)
**Service evaluation and audit**			
Leading	2 (22)	5 (50)	6 (55)
Informal support	1 (11)	6 (60)	5 (45)
Formal supervision	0	4 (40)	4 (36)
Member of governance committee	0	0	3 (27)
Not involved	3 (33)	1 (10)	1 (9)
**Quality improvement**			
Leading	4 (44)	2 (20)	1 (9)
Informal support	0	4 (40)	5 (45)
Formal supervision	0	3 (30)	1 (9)
Member of governance committee	0	0	2 (18)
Not involved	3 (33)	4 (40)	4 (36)

Response options not mutually exclusive

Twenty-two respondents (73%) had allocated research time through a research fellowship or grant. Three individuals (16% of those employed by the NHS) reported protected research time as part of their NHS job plan. This compared with four individuals with protected research time as part of their university job plan (36% of those employed in the university through any type of contract).

Twelve respondents (40%) had secured backfill to enable protected time for research. Another 12 reported that backfill was not applicable for their research activity and role because research was separate to any other duties. Of the 12 who had secured backfill, this was provided at the same clinical grade for six individuals, at a lower grade for five individuals, and one individual was unsure of the grade. Additionally, four respondents reported that no backfill funding was available, and one reported that their backfill funding was used as a cost-saving, rather than to appoint another person.

There were six additional comments regarding backfill (five from nurses/midwives and one from an allied health professional). Respondents covered all academic levels and were employed within the NHS or on joint or separate NHS/university contracts. Comments centred on the difficulty of recruiting to short-term, part-time roles that were often in a highly specialised clinical setting. Respondents recalled how this led to *“clinical work being absorbed by the current team”* (ID25), resulting in increased *“pressure on the service and other team members”* (ID15). It was identified that there was *“little acknowledgement within the clinical role of the contribution made by research work”* (ID35) and that research-active clinicians felt guilty because research was viewed as a *“foolish extravagancy when [the team] is so short staffed”* (ID28).

### Research capacity building

**Fig. 2:**
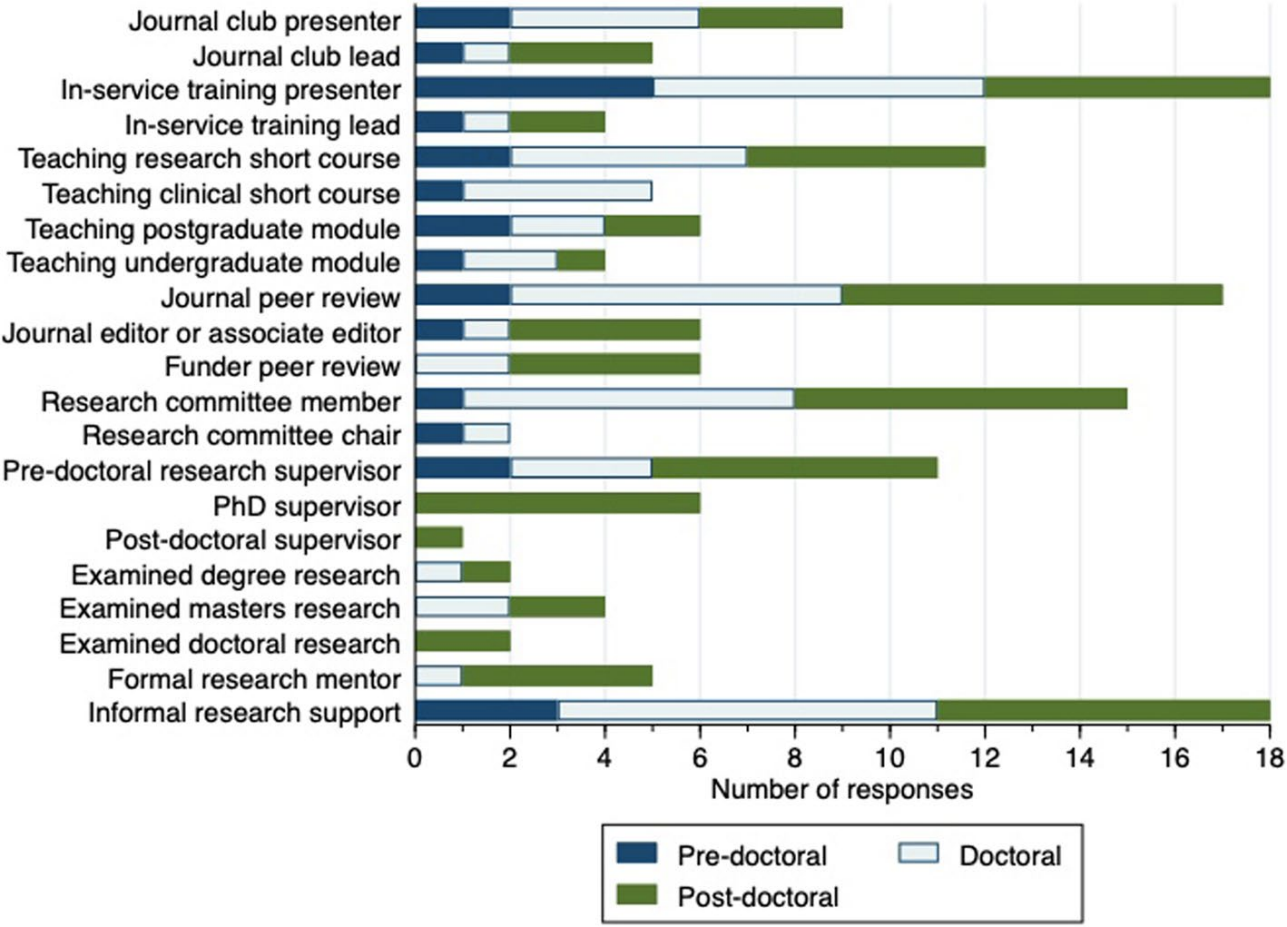
Reported research capacity building activities with last 12 months

Twenty-nine individuals completed the sections relating to research capacity building. Reported activities are shown in Fig. 2.

### Research into practice

Twenty respondents reported changes to their own clinical practice as a result of their research involvement in the previous 12 months. The most commonly reported changes were increased confidence in discussing treatment ambiguities with colleagues (12 individuals) and supporting their clinical service to change practice based on research (12 individuals; Table 5). Respondents were also asked about the broader impacts of their research across local, national, and international settings. Sixteen individuals identified changes to practice in these settings, the most common responses were new or different pathways of care at a local level (eight individuals) and the use of new or existing clinical guidelines at a national level (eight individuals). There were two additional comments; both highlighted potential future impacts of the individuals’ current research and mentioned that it was too early in the research process for research translation.

**Table 5:** Reported research translation activities leading to changes in clinical practice

	**Own practice** *n* = 30 (%)	**Locally**^**a**^ *n* = 30 (%)	**Nationally**^**b**^ *n* = 30 (%)	**Internationally**^**c**^ *n* = 30%
Use of clinical guidelines	9 (30)	5 (17)	8 (27)	0
Use of new or different assessment	6 (20)	2 (7)	2 (7)	2 (7)
Use of new or different patient reported outcome measures	4 (13)	3 (10)	0	0
Use of new or different treatments or interventions	7 (23)	5 (17)	2 (7)	0
Stopping provision of a test, treatment, or intervention	1 (3)	2 (7)	0	0
Introduction of, or changes in shared decision-making processes with patients	11 (37)	4 (13)	2 (7)	0
Supported changes in the format of care delivery	8 (27)	6 (20)	1 (3)	0
Changes in delivery of student placements	4 (13)	1 (3)	0	0
Changes in provision of clinical supervision	5 (17)	-	-	-
Increased confidence in discussing treatment ambiguities with patients	8 (27)	-	-	-
Increased confidence in discussing treatment ambiguities with colleagues	12 (40)	-	-	-
Deviation from existing clinical guidelines based on new research findings	2 (7)	-	-	-
Supported clinical service to change practice based on research	12 (40)	-	-	-
Not applicable, no clinical role	9 (30)	-	-	-
New or different pathways of care	-	8 (27)	1 (3)	0
Changes to staff training	-	6 (20)	0	0
No reported research translation	-	12 (40)	12 (40)	12 (40)
No response	1 (3)	2 (7)	2 (7)	2 (7)

Response options not mutually exclusive.—Question not asked. ^a^Locally – within own department, organisation or linked group of similarly located organisations; ^b^Nationally – across the UK or a whole geographic region within the UK; ^c^Internationally – outside the UK

### Patients and service users

**Fig. 3:**
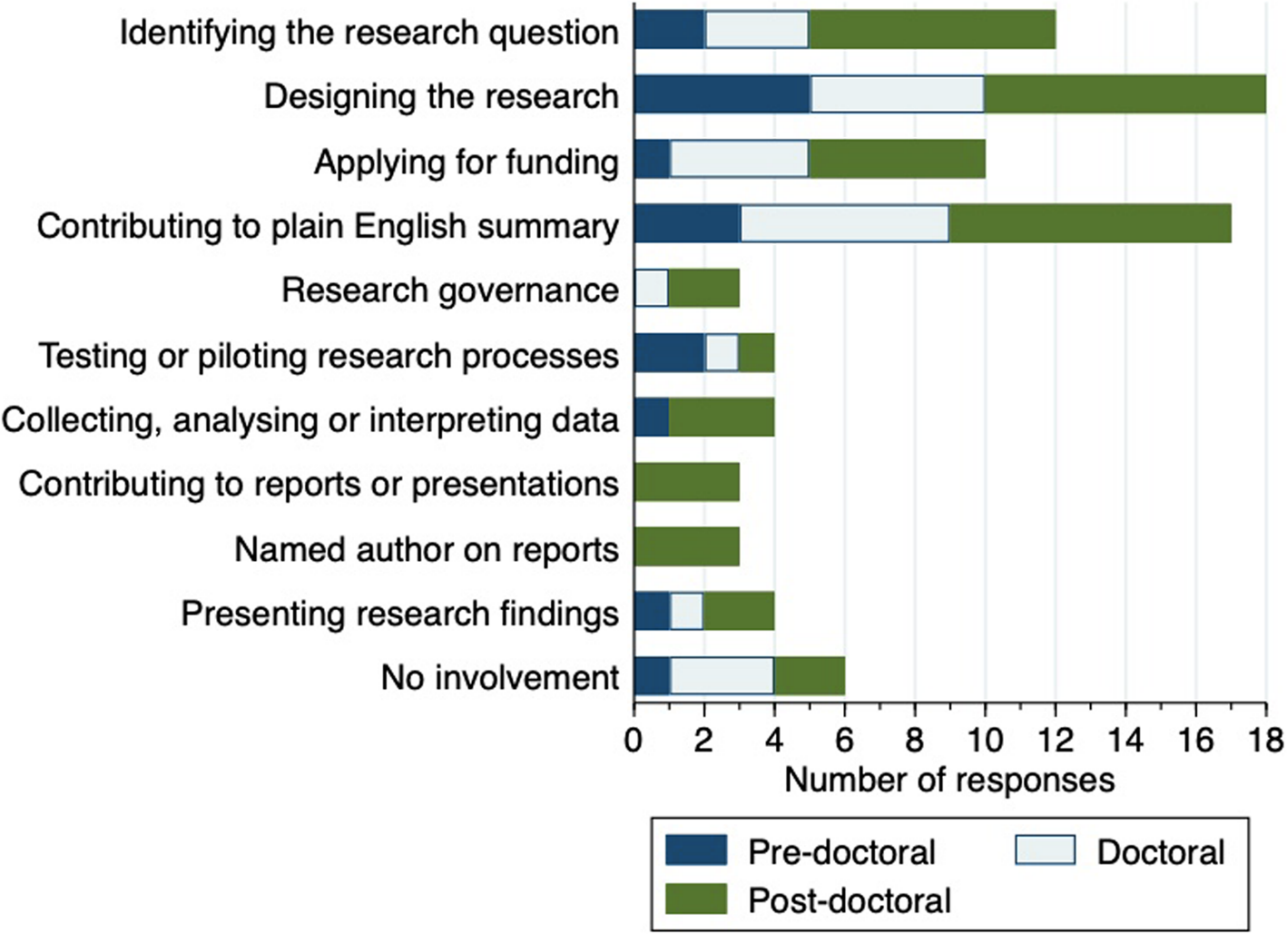
Patient and public involvement activities in the last 12 months

The majority of respondents reported involving patients and the public in their research. Two (7%) reported informal discussions and engagement activities, while 20 (67%) reported formal involvement through advisory groups and co-investigator roles. Involvement activities are shown in Fig. 3. Six respondents (20%) reported no patient or public involvement of patients or the public within their research activities.

Eight respondents (29%) provided their research participants with payment or a voucher, and six (21%) provided reimbursement for travel expenses. Of the 20 respondents who had formally involved patients or public in an advisory role, 10 (50%) provided payment or a voucher for this contribution, six (30%) provided travel or other expenses, while four (20%) did not provide any form of financial compensation.

Comments relating to the involvement of patients and the public focused on the beneficial impact of including different perspectives, for example, it was *“extremely important to help the study to be patient friendly and for constructive feedback”* (ID19) and *“securing PPI [patient and public involvement] from diverse communities is one of the great things going on in this organisation”* (ID28). However, respondents commented that it was difficult to set up payment for patient/public advisors within NHS systems *“payment of PPI group attendees is complicated… still figuring out how paying each person will work”* (ID15), or that they were advised to only offer expenses *“I was advised against payment/vouchers for participation or involvement, so the best I could offer was expenses”* (ID21).

Reported methods of sharing study findings with participants are outlined in Table 6. None of the respondents reported using a study website to share findings or providing a summary of any changes to practice. Only two respondents (both post-doctoral) reported updating their participant about any new research developments that arose from the existing project.

**Table 6:** Reported methods of updating research participants about study findings

	**Pre-doctoral** *n* = 9 (%)	**Doctoral** *n* = 10 (%)	**Post-doctoral** *n* = 11 (%)
Newsletters or email during the study	0	2 (20)	2 (18)
Study website	0	0	0
Summary of the findings at the end of the study	0	2 (20)	3 (27)
Updates of research outputs e.g. publications	1 (11)	4 (40)	3 (27)
Summary of any new research developments	0	0	2 (18)
Summary of any changes to practice	0	0	0
Presentations to participants or the public	1 (11)	1 (10)	3 (27)
Not applicable, no results yet	4 (44)	4 (40)	3 (27)
No response	3 (33)	1 (10)	1 (9)

Response options not mutually exclusive

### Research dissemination

**Fig. 4:**
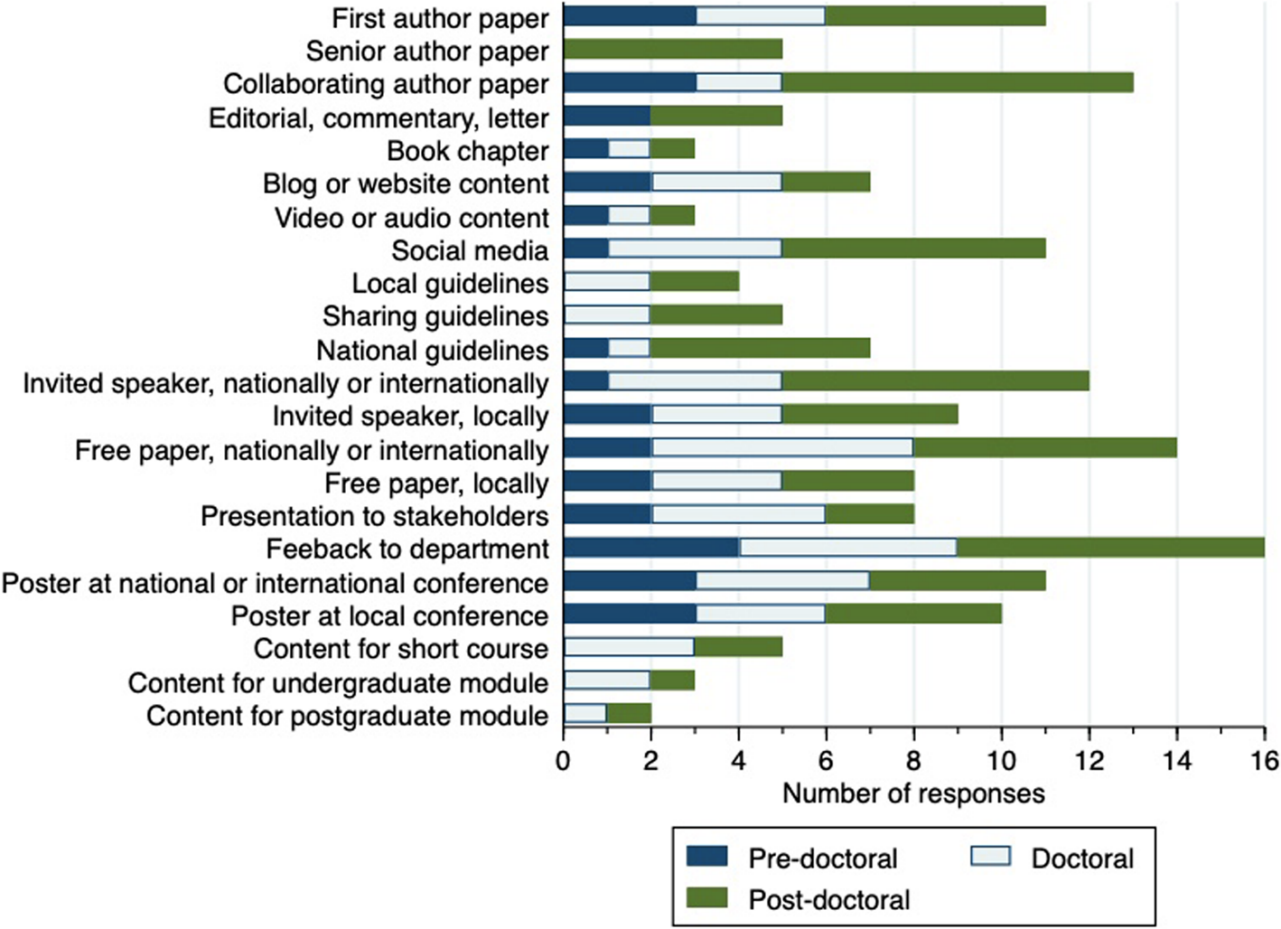
Reported research dissemination activities within the last 12 months

Twenty five respondents provided information about their research dissemination activities in the past 12 months (Fig. 4). The most commonly reported activities were providing feedback to their local department (16 individuals) and giving a presentation via a submitted abstract at a national or international conference (14 individuals).

Respondents were also asked about any prizes that they had been awarded in the past 12 months. Four had won prizes for poster presentations and four for conference free paper (oral) presentations. Two individuals had been awarded research-related prizes from their own organisation, one had been awarded fellowship of their professional body, and two had been awarded governmental honours.

### Economics and research funding

Two respondents (both pre-doctoral) reported cost-savings, improvement in efficiency or income generation as a result of their research activities. An additional 12 (40%) were unsure whether these impacts had occurred. Respondents were separately asked about the availability of support to carry out economic assessments; 17 (57%) reported that it was not relevant for their research; nine (30%) reported that they would have liked support but were unable to access it; and two (6%) reported that they had accessed either formal or informal support with health economic assessments.

Seven individuals provided additional comments regarding income generation and health economics. These focused on plans to run future workshops and teaching to generate income for the organisation (ID7, ID35); the idea that economic evaluation would occur at a later stage in their research (ID13, ID9, ID29); and not knowing where to start with health economics (ID30, ID28).

Twelve individuals reported applying for new research funding in the last 12 months. The outcomes of these applications are summarised in Table 7.

**Table 7:** Reported research funding applications in the previous 12 months

	**Applied (*n* = 12)**	**Awarded**	**Outcome unknown**	**Not awarded**
Local fellowship	2	1	0	1
National fellowship	7	5	2	1
Local research grant	1	0	1	0
National research grant, co-applicant	5	3	1	2
National research grant, lead applicant	5	2	2	2
International grant or fellowship	3	0	1	2
Commercial or industrial funding	1	0	1	0
External funding for research training	1	0	1	0
External funding for research dissemination	3	2	1	0
Other funding source	3	2	1	0

Response options not mutually exclusive; the same individual may have applied for more than one funding type within each category

### Collaborations

Twenty-two respondents reported that they had developed new research-related collaborations in the past 12 months. The nature of these collaborations is summarised in Table 8.

**Table 8:** New research-related collaborations developed in the previous 12 months

	**Pre-doctoral** ***n* = 9**	**Doctoral** ***n* = 10**	**Post-doctoral** ***n* = 11**
Different team in own healthcare organisation	3 (33)	4 (40)	5 (45)
Other healthcare organisation	3 (33)	3 (30)	6 (54)
Different team in own university	0	1 (10)	3 (27)
Other university	2 (22)	3 (30)	4 (36)
Patient group or support group	2 (22)	1 (10)	2 (18)
Local charity	1 (11)	1 (10)	0
National or international charity	1 (11)	2 (20)	0
Professional body	1 (11)	3 (30)	2 (18)
National government	0	1 (10)	1 (9)
Industry or other commercial company	0	1 (10)	2 (18)
No new collaborations	2 (22)	0	1 (9)
No response	3 (33)	1 (10)	1 (9)

Response options not mutually exclusive

## Discussion

The first aim of this project was to develop a framework to illustrate the key impacts of NMAHPP research activity as identified by different stakeholder groups. This framework was informed by the existing literature, and refined with feedback from research participants, research-active NMAHPPs and healthcare managers, using an established framework development methodology [31]. Eight elements were included, each representing a distinct aspect of research impact: clinical background, research and service improvement activities, research capacity building, research into practice, patients and service users, research dissemination, economics and research funding, and collaborations. These elements were accepted by all stakeholders and were anticipated to reflect tangible benefits within the healthcare environment. There are areas of overlap with existing research impact frameworks [40], however to the best of our knowledge, this is the first research impact framework with specific relevance for NMAHPP clinicians.

The second aim of this project was to create and pilot a research impact capture tool that could be used to document the breadth of impacts associated with each element of the framework. The impact capture tool was developed with dual functionality. The first function was to provide information at the organisation level which could be used to evidence the collective impacts of existing research activity and monitor change over time. There has been a recent drive to support clinical academic opportunities for NMAHPPs in the UK [2, 3, 21], however there is currently no agreed method of recording the impacts of clinical academic activity. We present our research impact capture tool as a starting point that is inclusive of all NMAHPP disciplines.

The second function of the research impact capture tool was to provide a means for individual research-active clinicians to record and discuss their research activity, outputs and impact, in a standardised way as part of annual appraisals. This supports the ethos that research is everyone’s business [29] and encourages recognition of research-related achievements.

Existing UK strategies for recording and measuring research impact include the Research Excellence Framework (REF) [41]. This is a university-based metric which is used to generate league tables that inform the allocation of future research funding. The REF primarily focuses on academic impacts, including the quality of publications and the local research environment, although individual impact case studies now account for approximately a fifth of the REF score [41]. Anecdotally, these case studies are often developed retrospectively in the absence of robust data on the various impacts across the lifecourse of the research programme. Importantly, REF only applies to university-based, rather than NHS-based researchers; REF scores are not released at the individual level; and universities submit applications within pre-defined units of assessment, meaning that the smaller NMAHPP disciplines may be overlooked.

Other approaches explore research impact at the level of an individual research study, for example VICTOR (making VIsible the ImpaCT Of Research) [42]. This is a qualitative reflection of impact, which is valuable for the individual research team, but difficult to compare across studies, departments and organisations, or over time. Furthermore, it does not include aspects related to recruitment and retention of clinical academic staff or career aspirations, which is very important within the clinical setting [43].

The results of our research impact capture tool pilot demonstrate that this is a feasible method of collecting information that is useful at the individual and organisational level. We did not measure the duration of completion, but initial testers reported 15–25 min depending on the use of the additional free text boxes.

We chose to present data stratified by academic level (pre-doctoral, doctoral, and post-doctoral) with the expectation that some of the elements of the impact framework might differ across these groups [27]. Some research activities and impacts were more prevalent among post-doctoral respondents, for example developing and sharing clinical guidelines, being an invited conference speaker, authoring publications, providing material for educational courses, developing new collaborations and involvement in research governance committees. Post-doctoral respondents also incorporated more patient and public involvement activities as part of their research. This shows the added value that these individuals bring to their teams and organisations. However, there are currently few opportunities for post-doctoral NMAHPP clinicians to combine research and clinical practice [13, 36, 44, 45].

The most common research capacity building activities across all academic levels were informal research support, presenting at in-service training sessions and journal peer review. Informal support is likely to be an important factor in encouraging colleagues to become research aware and research active thereby strengthening research culture and capacity [27, 33, 44]. Having access to colleagues who can answer questions about research or other service improvement activities, and provide constructive feedback on plans and proposals, is likely to create positive impacts for less experienced researchers. This informal support may previously have gone unrecognised without a means of documenting this input. In our experience, all three of these activities (informal research support, presenting at in-service training sessions and journal peer review) are often carried out in NMAHPP clinicians’ own time, falling outside the remit of their allocated clinical or research time. This may therefore be an additional burden for research-active clinicians.

More than half of respondents reported translation of their research into wider clinical practice within the previous 12 months. Being embedded in clinical service may support more rapid research translation for several reasons. Firstly, research-active clinicians, supported by appropriate patient and public involvement, are well placed to identify research questions that are truly relevant to practice. Secondly research-active clinicians are likely to have credibility among their peers, plus established multidisciplinary networks to support research dissemination and implementation. The often quoted statistic that it takes 17 years between research completion and implementation in practice may need revising in the context of NMAHPP practice-based research [46]. However, we appreciate that many of the reported research translation activities occurred in local or national settings and were predominantly the development and use of clinical guidelines. The relationship between NMAHPP research activity and the implementation of research findings into routine clinical practice warrants further exploration.

Importantly, the majority of respondents across all clinical grades, academic levels and clinical backgrounds held career aspirations for a clinical academic role within the NHS. Such roles are neither clearly defined [47], nor widely available in the UK [27, 44]. Three individuals in our current sample reported that they had joint clinical academic contracts between university and NHS, but it was not possible to identify whether these were formally established roles, temporary posts associated with funding from research grants or fellowships, or misinterpretation of the term ‘joint contract’. We plan to provide more detailed explanation of the different contract types for the next iteration of the impact capture tool. Where clinical academic roles for NMAHPPs are reported in the literature, they often appear to have been created for specific individuals rather than as accessible career pathways [19, 20]. Similarly, in our sample, very few participants (*n* = 3) had protected time for research as part of an NHS job plan. Those who did were at a post-doctoral level and were also supported by research grants or fellowships.

Access to research opportunities, including research funding, was reported as a key factor in both recruitment and retention across our sample. This has also been reported elsewhere [26, 27, 48]. At a time when the NHS is struggling with staffing [43, 49], the opportunity to access clinical academic career pathways may enhance retention of experienced staff who create positive impacts for patient care. In addition, these key staff members and the opportunities available may attract others to the institution. Nearly half of our current sample reported that individuals had joined their team in the past year chiefly because of the recognised research culture and capability.

We recorded a range of honorary contract nomenclature, with no clear structure or comparison between these roles. We suggest that a standardised hierarchy should be established for NMAHPP professionals with the aim of achieving parity based on clinical and academic experience. This applies to both honorary contracts within the health service and academia and would be equivalent to the structure used for clinical doctors [50, 51].

### Limitations

Our research impact capture tool has several important limitations. Firstly, it relies on self-reported data. However, this is a common format for other systems of recording impact, including REF, ResearchFish® and VICTOR [42]. The timing of data collection needs to be carefully considered to avoid potential survey fatigue. Our pilot period coincided with the NHS Staff Survey, which has organisation- and department-level targets for completion [52]. Despite this, we achieved a response rate of > 50%, illustrating high levels of engagement. However, we plan to move the completion window for the next round to avoid this overlap.

Secondly, our sample population was not fully representative of the wider population of research-active NMAHPPs within the Trust and affiliated organisations. The self-selected sample may have been biased towards those who were interested in capturing the impact of research activity, especially due to the limited opportunities currently available for those wishing to pursue a clinical academic career in the UK. Therefore, the reported findings may over-estimate the impacts of research activity compared with a wider population. Additionally, we had no responses from healthcare scientists or psychologists, only one respondent was male, and there was limited ethnic diversity. We will further refine our sampling strategy and engagement activities for subsequent rounds to ensure that all NMAHPP disciplines are aware of the purposes of the impact capture tool and their inclusion within the NMAHPP acronym. The lack of ethnic diversity in our sample may also be representative of wider patterns of limited diversity within healthcare disciplines, for example in 2022, only 12% of allied health professionals came from Black, Asian or Minority Ethnic (BAME) backgrounds compared with 20% across the NHS workforce [53]. Initiatives such as FAIResearch have been established to support fair, accessible, inclusive research opportunities for allied health professionals [54], and demographic data from the research impact capture tool could be used to record this aspect of research involvement.

Thirdly, there is currently no method to link the data captured from this tool with measures of experience or feedback from patient and public advisors or research participants. A possible future expansion could include direct data capture or data triangulation with participants or other public contributors.

Finally, we did not collect data about individual departments within employing organisations. This would help identify multidisciplinary areas of research collaboration and is something we plan to add for the next iteration. This will allow learning from research-active departments and enable additional support to be provided for areas with lower research involvement or impact.

## Conclusion

In conclusion, we used established framework development methodology to create a research impact framework specifically for NMAHPP research within clinical practice. Through ongoing stakeholder feedback, this framework was used to build and pilot a series of questions to capture the impact of each framework element. We report our preliminary findings as a demonstration of these impacts in relation to research-active NMAHPPs within Imperial College Healthcare NHS Trust and associated organisations. We encourage other organisations to collaboratively use, refine and share our impact capture tool, with the aim of developing a standardised method of recording and reporting the impacts of research activity by NMAHPP clinicians. This will enable NMAHPP research leaders to record changes over time within an organisation or after implementation of an intervention aimed at supporting and increasing NMAHPP research activity. Additionally, individual research-active clinicians can use the information for their annual appraisal, further promoting the impacts of local research activity to clinical line managers, and ensuring that the research-active clinicians’ contributions are recorded and recognised.

## Supplementary Information

**Additional file 1.**

## Data Availability

Datasets from studies that informed development of the research impact capture framework are available from the Open Science Framework (https://osf.io/s5n3d/ and https://osf.io/wurz3/) and ReShare Repository (https://reshare.ukdataservice.ac.uk/855956/). The pilot research impact capture tool generated as part of the current study is available from the Open Science Framework (https://osf.io/57vwn/). All new summary data generated and analysed during the current study are included in this published article.

## Abbreviations

CARC: Clinical Academic Research Committee
CARIN: Clinical Academic Research Careers Implementation Network
NHS: National Health Service
NMAHPP: Nursing, Midwifery, Allied health professions, Healthcare science, Pharmacy, Psychology
PAG: Patient and public Advisory Group
PGRF: PostGraduate Research Forum
REF: Research Excellence Framework
UK: United Kingdom
VICTOR: Making Visible the ImpaCT of Research
