# Supplementary material for: Development of a framework and research impact capture tool for nursing, midwifery, allied health professions, healthcare science, pharmacy and psychology (NMAHPPs)
Source: BMC Health Serv Res. 2023 May 3;23:433. doi: 10.1186/s12913-023-09451-2 (PMC10157965; doi:10.1186/s12913-023-09451-2)
Supplement: Supplementary file 1 — Additional file 1. [file 12913_2023_9451_MOESM1_ESM.pdf]

Appendix 1: List of UK Allied Health Professions

Art therapists

Dieticians

Drama therapists

Music therapists

Occupational therapists

Operating department practitioners

Orthoptists

Osteopaths

Paramedics

Physiotherapists

Podiatrists / chiropodists

Prosthetists and orthotists

Radiographers

Speech and language therapists

Source: NHS England 'The 14 allied health professions' https://www.england.nhs.uk/ahp/role/. Accessed 13/12/2022
